# Supplementary material for: Lung Ultrasound Patterns and Clinical-Laboratory Correlates during COVID-19 Pneumonia: A Retrospective Study from North East Italy
Source: J Clin Med. 2021 Mar 20;10(6):1288. doi: 10.3390/jcm10061288 (PMC8004000; doi:10.3390/jcm10061288)
Supplement: Supplementary file 1 [file jcm-10-01288-s001.pdf]

Table S1. Chest landmarks for the LUS protocol.

Anterior	Right	R1: upper on the midclavicular line above the internipple line
		R2: basal on the midclavicular line below the internipple line
	Left	L1: upper on the midclavicular line above the internipple line
		L2: basal on the midclavicular line below the internipple line
Lateral	Right	R3: upper on the midaxillary line above the internipple line
		R4: basal on the midaxillary line below the internipple line
	Left	L3: upper on the midaxillary line above the internipple line
		L4: basal on the midaxillary line below the internipple line
Posterior	Right	R5: upper on the paravertebral line above the inferior angle of shoulder blade line
		R6: basal on the paravertebral line below the inferior angle of shoulder blade line
	Left	L5: upper on the paravertebral line above the inferior angle of shoulder blade line
		L6: basal on the paravertebral line below the inferior angle of shoulder blade line

Table S2. LUS scoring.

B-lines	0	No B-lines. A-lines (normal reverberation artifacts of the pleural line that when accompanied by lung sliding correspond to normal aeration of the lung)
	1	A-B lines
	2	B-lines (hyperechoic lines vertical to the pleura line, arising from it and reaching the edge of the screen erasing A-lines, which represent rever- beration artifact through edematous interlobular septa or alveoli)
	3	White lung (coalescent B-lines that correspond to severe lung aeration loss)
Lung consolidation	0	No consolidation
	1	Small subpleurical consolidation
	2	Low-grade lung hepatization
	3	High-grade lung hepatization
Pleural line integrity	0	Intact pleural line
	1	Pleural thickening
	2	Irregular pleural line
	3	Interrupted pleural line
Pleural effusion	0	No pleural effusion
	1	Low-grade pleural effusion
	2	Medium-grade pleural effusion
	3	High-grade pleural effusion
